# Pretreatment Primary Tumor Stage is a Risk Factor for Recurrence in Patients with Esophageal Squamous Cell Carcinoma Who Achieve Pathological Complete Response After Neoadjuvant Chemoradiotherapy

**DOI:** 10.1245/s10434-020-09219-6

**Published:** 2020-10-19

**Authors:** Roberta La Mendola, Maria Bencivenga, Lorena Torroni, Luca Alberti, Michele Sacco, Francesco Casella, Cecilia Ridolfi, Nicola Simoni, Renato Micera, Michele Pavarana, Giuseppe Verlato, Simone Giacopuzzi

**Affiliations:** 1 General and Upper GI Surgery Division, University of Verona, Verona, Italy; 2 Unit of Epidemiology and Medical Statistics, Department of Diagnostics and Public Health, University of Verona, Verona, Italy; 3 Unit of Radiotherapy, Verona University Hospital, Verona, Italy; 4 Unit of Medical Oncology, Verona University Hospital, Verona, Italy

## Abstract

**Background:**

Although pathological complete response (pCR) after multimodal treatment for esophageal cancer is associated to the best prognosis, recurrence may occur in 20–40% of cases. The present study investigated the recurrence pattern and predictive factors of recurrence after pCR in patients with esophageal cancer.

**Methods:**

In this study, 427 patients received preoperative treatment for either esophageal squamous cell carcinoma (SCC) or adenocarcinoma at Verona University Hospital between 2000 and 2018. Of these, 145 patients (34%) achieved a pCR. Long-term prognosis, recurrence pattern, and risk factors for relapse in pCR patients were analysed.

**Results:**

During a median follow-up of 52 months, 37 relapses (25.5%) occurred, mostly at distant level (*n* = 28). Nearly all locoregional relapses (8/9) were detected in SCC cases. The 5-year overall survival and cancer-related survival were 71.7% (95% confidence interval [CI] 62.6–78.9%) and 77.5% (95% CI 68.5–84.2%) respectively. Male sex, higher body mass index, and cT4 were significant risk factors for recurrence at univariate analysis. The multivariate analysis confirmed the role of cT4 as predictor of recurrence only in SCCs.

**Conclusions:**

Esophageal cancer recurs in about one-fourth of pCR cases. A fair number of local recurrences occurs in SCCs, but the main problem is the systemic disease control. According to our analysis, SCCs patients with cT4 stage have an increased risk to recur, so they should be managed differently by a personalized approach in terms of adjuvant treatment and follow-up.

During the past two decades, neoadjuvant chemoradiotherapy followed by surgery was established as the standard of care in patients. Locally advanced esophageal cancer (EC) was associated with a higher rate of radical (R0) resections and better overall survival (OS) and cancer-related survival (CRS) compared with surgery alone.^1^,^2^ Preoperative treatments allow a pathological complete response (pCR) to be obtained, defined as the total regression of the tumor both at the site of the primary lesion and at the removed lymph nodes (ypT0N0), in approximately 45% of SCCs and 25% of ACs.^1^,^3^ The newborn category of patients with pCR has the best prognosis among locally advanced EC cases. Indeed, there is a clear correlation between the response to neoadjuvant treatment and long-term survival, which is reported to be higher than 50% at 5 years in pCR regardless of histology.^4^–^9^

However, 20–31% of pCR patients develop cancer recurrence, often in the first 2 years after treatment.^6^,^10^ The problem of locoregional and distant relapses of ypT0N0 esophageal and esophagogastric cancers is a highly debated topic. Many authors are currently analysing the factors associated with this unexpected event. Predictive factors of recurrence are mostly represented by clinical and pathological characteristics, suggesting that the category of pCR includes neoplasms with different features, which significantly affect the prognosis and which should guide the postoperative strategies and follow-up.

Our study fits into this context: to describe the location and timing of recurrence in a sample of patients with pCR after neoadjuvant treatment and surgery for EC and to identify the possible predictive factors.

## Methods

### Patients and Diagnostic Workup

We performed a retrospective analysis on our database of 599 patients who underwent radical surgery for EC from 2000 to 2018 at our institution. Among them, we selected both SCC and AC (Siewert type I and II) who were treated by neoadjuvant chemoradiotherapy reaching a pCR. Demographic, clinical, pathological, and surgical data were recorded, including Charlson Comorbidity Index (CCI).

All patients had their diagnosis after an upper gastrointestinal endoscopy with histological confirmation of EC. They were staged with endoscopic ultrasound (EUS), thoracic, and abdominal contrast computed tomography (CT) scan and whole body fluorodeoxyglucose-positron emission tomography (18F-FDG PET). Moreover, neck contrast CT scan and ultrasonography, bronchoscopy, and magnetic resonance imaging (MRI) of esophagus were performed in SCC cases. EC was clinically staged according to the eighth edition of the American Joint Committee on Cancer (AJCC) classification.^11^ Neoadjuvant therapy was indicated for any patient with a clinical T2-4 and/or TxN + EC without contraindications to treatment. Longitudinal extension of the tumor was measured during preoperative endoscopy or intraoperatively before resection.

### Neoadjuvant Treatment

Most patients included in the present series had a preoperative radiochemotherapy according to the protocol proposed at our institution.^12^ It is characterized by a first phase of induction chemotherapy (3 cycles) with docetaxel, cisplatin, and 5-fluorouracil (TCF), followed by a second step of concurrent radiochemotherapy with the same drugs for 5 cycles plus 50.4 Gy. Some cases of Siewert type II AC with a preponderant abdominal extension were addressed to perioperative chemotherapy by the multidisciplinary team. We recorded all the complications of treatment with subsequent dose reduction or discontinuation.

### Surgery

Surgery was performed 6–8 weeks after the end of the radiochemotherapy and 4–5 weeks after the end of chemotherapy. The extent of surgical resection was selected according to primary tumor and nodal site, as well as patients’ performance status. ECs located above the carina were treated by McKeown esophagectomy with cervical anastomosis. Lower thoracic ECs and cardia cancers (Siewert type I and II with limited gastric invasion) underwent Ivor-Lewis esophagectomy with intrathoracic anastomosis. Some cases of Siewert type II AC with prevalent abdominal extension were managed by total gastrectomy with Roux-en-Y esophagojejunostomy. Primary lesion resection was always associated to a lymphadenectomy. In case of total gastrectomy and transhiatal esophagectomy, a D2 lymphadenectomy extended to lower mediastinal nodes was usually performed. D2 dissection was sometimes associated to the removal or para-aortic nodes. In case of Ivor-Lewis esophagectomy, a D2 abdominal and a mediastinal standard dissection was the lymphadenectomy of choice, whereas in case of McKeown esophagectomy, a D2 abdominal and an extended thoracic lymphadenectomy was usually achieved.

All surgical complications were recorded and classified according Clavien-Dindo scale.^13^ In particular, “major” complications were defined by a grade higher than or equal to IIIa.

### Histopathological Analysis

Paraffin-embedded, formalin-fixed pathological slides from surgical specimens were stained with hematoxylin and eosin or alcian blue and sometimes examined by immunohistochemistry. In particular, macroscopic lesions, resection margins, and resected lymph nodes were analysed.

Tumor staging refers to the eighth edition of AJCC classification.^11^ Evaluation of tumor regression grade (TRG) according to Mandard classification^14^ also was performed. pCR was defined as the complete regression of primary lesion and nodal metastases, as previously described by our group.^9^ Evidence of residual tumor either in the primary site or in the lymph nodes represents an exclusion criterion for our study.

### Follow-Up

There are no clear guidelines about follow-up in EC. At our Institution, an outpatient clinical evaluation is usually scheduled 4 months after surgery and then every 6 months for the first 5 years. After that, patients are evaluated annually for another 5 years. Our follow-up schedule includes laboratory tests (cell blood count, tumor markers), an upper gastrointestinal (GI) endoscopy, and a thoracoabdominal contrast CT scan. Surveillance in SCC includes bronchoscopy and neck CT and ultrasound scan.

Disease recurrence was always detected by follow-up examinations. In selected cases, it was confirmed by biopsy. Time to relapse was considered to be the interval from surgery to recurrence diagnosis. Median follow-up was 52 (range: 25.5–95.5) months.

### Statistical Analysis

Quantitative variables were expressed as the median and interquartile range, and qualitative variables as percentages. Significance of differences was evaluated by the Mann–Whitney test for quantitative variables and Fisher’s exact test for categorical variables.

Survival, expressed as OS and CRS, was estimated by the Kaplan–Meier method. Statistical significance of differences among groups was assessed by the log-rank test. All deaths were considered as events in OS. In cancer-related survival, only deaths from cancer recurrence were taken into account, whereas deaths from other causes were considered as censored observation at the time of occurrence. Risk factors for recurrence-related deaths were further investigated in multivariable Cox regression model. The strength of the association was expressed by the hazard ratio (HR) with 95% confidence interval (CI). Significance of differences was evaluated by the Wald test. The assumption of proportional hazards over time was verified by graphic methods: log(-log(survival)) was plotted versus log(time), and it was verified whether the graph resulted in parallel curves.

Statistical significance was set at *p* value < 0.05, and analyses were performed with the statistical software STATA^®^ version 16.0 (StataCorp, College Station, TX).

## Results

### Description of Patient Characteristics

Among the 599 patients who underwent esophagectomy for esophageal SCC or AC, 427 received preoperative treatment, and pCR was achieved in 145 cases (34%) The pCR rate was (85/195) 43.6% in SCC and 25.9% (60/232) in AC. One patient lost to follow-up was excluded from the analysis, as well as three other patients who had presented metastases at diagnosis, although later achieved pathological complete response at the primary site. Clinical and surgical characteristics of our series are summarized in Table 1.

**Table 1:** Main demographic and clinical characteristics of patients with pCR, compared by histotype

Characteristic	Overall (*N* = 145)	AC (*N* = 60)	SCC (*N* = 85)	*p* value
Sex				0.085
M	118 (81.4%)	53 (88.3%)	65 (76.5%)	
F	27 (18.6%)	7 (11.7%)	20 (23.5%)	
Age (median, p25–p75)	64 (58–69)	64 (59.5–68.5)	63 (57–68)	0.378
BMI (median, p25–p75)**	23.87 (21.3–27.8)	26.96 (22.6–29.3)	22.40 (20.2–25.4)	< 0.001*
Normal weight	63 (60.0)	17(40.5)	46 (73.0)	0.001*
Overweight/obese	42(40.0)	25 (59.5)	17 (27.0)	
CCI				0.207
≤ 3	18 (12.4%)	9 (15.0%)	9 (10.6%)	
4–5	78 (53.8%)	27 (45.0%)	51 (60.0%)	
> 5	49 (33.8%)	24 (40.0%)	25 (29.4%)	
Tumor length (cm)**				0.301
< 2	9 (7.8%)	2 (4.3%)	7 (10.3%)	
2–5	70 (60.9%)	27 (57.5%)	43 (63.2%)	
> 5	36 (31.3%)	18 (37.3%)	18 (26.5%)	
Clinical stage**				0.156
II	53 (39.3%)	27 (46.5%)	26 (33.8%)	
III	82 (60.7%)	31 (53.5%)	51 (66.2%)	
cT				0.119
1	1 (0.7%)	0	1 (1.3%)	
2	30 (22.2%)	14 (24.1%)	16 (20.8%)	
3	85 (63.0%)	40 (69.0%)	45 (58.4%)	
4a/b	19 (14.1%)	4 (6.9%)	15 (19.5%)	
cN				0.175
0	36 (26.7%)	19 (32.8%)	17 (22.1%)	
+	99 (73.3%)	39 (67.2%)	60 (77.9%)	
cM				–
0	135 (100%)	58 (43%)	77 (57.0%)	
Neoadjuvant treatment				0.028*
CRT	141 (97.2%)	56 (93.3%)	85 (100%)	
CT	4 (2.8%)	4 (6.7%)	0	
Treatment complications (yes)	21 (14.2%)	9 (15.0%)	12 (14.1%)	1.000
Surgery				< 0.001*
IL esophagectomy	85 (58.6%)	49 (81.7%)	36 (42.4%)	
McK esophagectomy	41 (28.3%)	4 (6.7%)	37 (43.5%)	
TG	4 (2.8%)	3 (5.0%)	1 (1.2%)	
Other	15 (10.3%)	4 (6.7%)	11 (12.9%)	
Retrieved lymph nodes				0.678
< 15	30 (21.1%)	14 (23.3%)	16 (19.5%)	
≥ 15	112 (78.9%)	46 (76.7%)	66 (80.5%)	
Surgical complications (yes)	59 (40.7%)	24 (40.0%)	35 (41.2%)	1.000
Major surgical complications	37 (62.7%)	14 (58.3%)	23 (65.7%)	0.594

*AC* adenocarcinoma, *SCC* squamous cell carcinoma, *BMI* body mass index, *CCI* Charlson Comorbidity Index, *CRT* chemoradiotherapy, *CT* chemotherapy, *IL* Ivor Lewis, *McK* McKeown, *TG* total gastrectomy

Major surgical complications: ≥ IIIa according to Clavien-Dindo classification

*Statistically significant

**Information on BMI, tumor length, and clinical stage was missing in 40, 30, and 10 patients respectively

Eighty-one percent of our series was male. Median age was 64 (range: 58–69) years, and the median body mass index (BMI) was 23.9 (range: 21.3–27.8) kg/m^2^. Of the patients examined, 97.3% underwent neoadjuvant chemoradiotherapy. Only four patients received chemotherapy only (2.7%); 85.5% of patients did not develop preoperative treatment complications needing underdosing or interruption. In addition, the type of surgery performed, the number of lymph nodes removed (< 15 vs. ≥ 15), and surgical complications were recorded.

Eighty-five (58.6%) of ECs analysed were SCC, and 60 (41.4%) were AC. In our sample, patients with the two major histotypes showed homogeneous clinicopathological characteristics, differing significantly only for BMI that was higher in AC group, and for type of surgery as total gastrectomy and McKeown esophagectomy were almost exclusively performed respectively in AC and SCC patients.

### Recurrence Pattern

Thirty-seven recurrences occurred during the follow-up, corresponding to a cumulative incidence of 25.5% (95% CI 18.6–33.4%). Incidence of recurrence was similar in AC (15/60 = 25%) and SCC (22/85 = 25.8%) tumors. Most recurrences were distant (75.7%), whereas nine (24.3%) were exclusively locoregional. Site of recurrence varied as function of histotype, as nearly all locoregional recurrences were observed in the SCC group (*n* = 8), with only one occurring in the AC group (*p* = 0.056; Table 2a).

**Table 2:** a Recurrence pattern by site and histotype; *p* = 0.056. b Recurrence pattern by time and histotype; *p* = 0.049

a				b			
Recurrence site	Overall (*N* = 37)	AC (*N* = 15)	SCC (*N* = 22)	Recurrence time (mo)	Overall (*N* = 36)	AC (*N* = 15)	SCC (*N* = 21)
Locoregional	9 (24.32%)	1 (6.67%)	8 (36.36%)	≤ 12	19 (52.8%)	11 (73.3%)	8 (38.1%)
Systemic	28 (75.68%)	14 (93.33%)	14 (63.64%)	> 12	17 (47.2%)	4 (26.7%)	13 (61.9%)

*AC* adenocarcinoma, *SCC* squamous cell carcinoma

We also classified relapses into early (occurring within a year of surgery) and late (occurring later than a year after surgery). The time of recurrence was missing for one patient; therefore, this analysis was performed on 36 patients. Nineteen cases (52.8%) occurred within a year. The remaining 17 (47.2%) relapsed later. Of note, we found that all early metastases but two were distant. Interestingly, all brain metastases, which we recorded in five patients, occurred within 1 year after surgery. With regard to the timing of recurrence, SCCs tended to relapse later compared with AC (*p* = 0.049; Table 2b). The median time of recurrence was 12 months (interquartile range: 7.5–31). In particular, the most delayed recurrence occurred after 42 months in AC and after 72 months in SCC cases, and they were both systemic.

### Survival

**Fig. 1:**
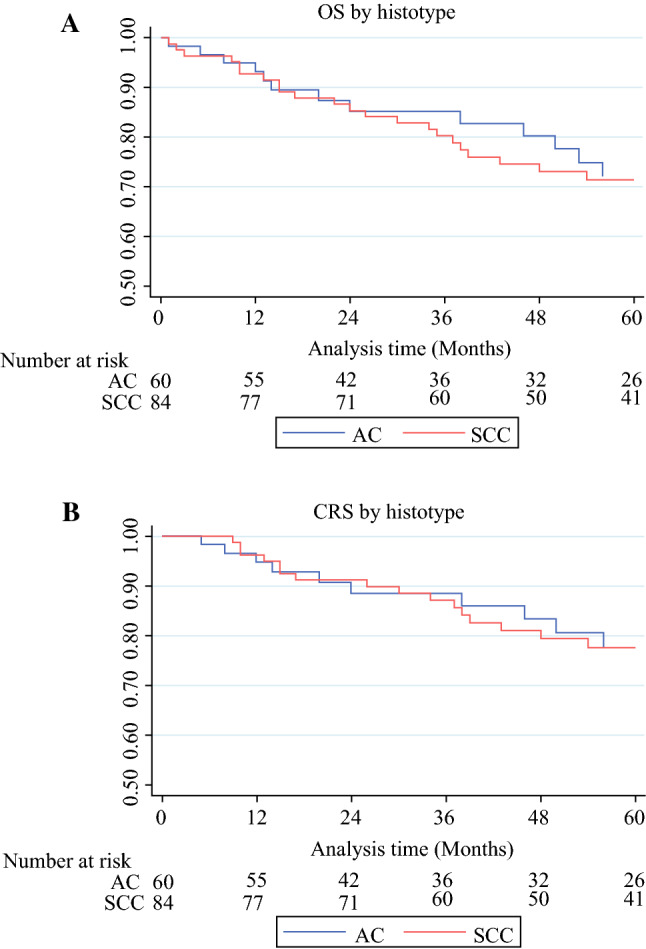
**a** Overall survival (OS) of pCR patients by histotype; *p* = 0.752. **b** CRS of pCR patients by histotype; *p* = 0.982. *pCR* pathological complete response, *AC* adenocarcinoma, *SCC* squamous cell carcinoma, *CRS* cancer-related survival

**Fig. 2:**
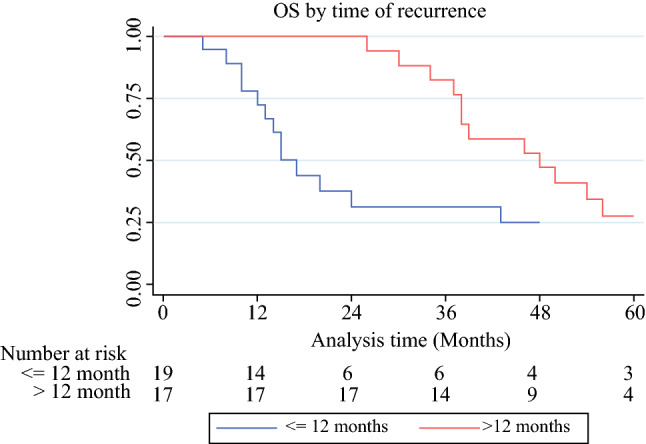
Overall survival (OS) of pCR-relapsed patients by time of recurrence; *p* < 0.001 (0.0042) *pCR* pathological complete response

The 5-year OS and CRS of our patients with pCR were respectively 71.7% (95% CI 62.6–78.9%) and 77.5% (95% CI 68.5–84.2%), with a similar trend for SCCs and ACs (Fig. 1a, b). Five-year OS decreased to 25.1% (95% CI 8.0–46.9%) and 27.5% (95% CI 9.0–49.9%) in patients with early and late relapses, respectively. As expected, OS of early relapsed patients was significantly worse than those with a late recurrence (*p* = 0.0042; Fig. 2).

### Predictive Factors of Recurrence

In univariable survival analysis sex, BMI and cT were significantly associated with disease recurrence in the whole series; prognosis was worse in men, overweight, and cT4a/b patients than in women, normal weight, and cT1/cT2/cT3. In subgroup analysis, sex and cT remained significant predictive factors among SCC patients, whereas neoadjuvant treatment complications emerged as prognostic factors among AC patients (Table 3). Considering that the observed recurrence pattern was different between SCC and AC and the risk factors for recurrence also completely differed between them in the univariate analysis, we decided to perform separate multivariate analysis for SCC and AC.

**Table 3:** Univariate analysis for recurrence risk factors in pCR population, compared by histotype

	Overall (*N* = 144)			AC (*N* = 60)			SCC (*N* = 84)		
	Case/total	5-year CRS (95% CI)	*p*	Case/total	5-year CRS (95% CI)	*p*	Case/total	5–year CRS (95% CI)	*p*
Sex			0.009*			0.301			0.020*
Men	35/117	72.9% (62.6–80.8%)		14/53	75.7% (59.1–86.4%)		21/64	70.9% (56.8–81.2%)	
Women	2/27	1.00		1/7	1.00		1/20	1.00	
BMI			0.026*			0.139			0.344
Normal weight	12/63	84.9% (70.6–92.6%)		2/17	88.9% (43.3–98.4%)		10/46	84.7% (68.9–92.9%)	
Overweight	15/42	65.6% (46.7–79.2%)		11/25	59.6% (33.9–78.1%)		4/17	73.7% (43.5–89.5%)	
CCI			0.456			0.868			0.346
≤ 3	2/18	87.4% (58.1–96.7%)		1/9	88.9% (43.3–98.4%)		1/9	87.5% (38.7–98.1%)	
4–5	24/78	73.8% (60.5–83.2%)		7/27	75.5% (46.7–90.1%)		17/51	72.5% (56.4–83.5%)	
> 5	11/48	79.8% (63.1–89.5%)		7/24	74.9% (49.1–88.9%)		4/24	84.4% (58.2–94.8%)	
Tumor length (cm)			0.527			0.192			0.789
< 2	4/9	77.8% (36.5–93.9%)		1/2	50.0% (0.6–91.0%)		3/7	85.7% (33.4–97.9%)	
2–5	20/70	76.1% (61.9–85.6%)		7/27	83.7% (55.7–94.8%)		13/43	71.9% (53.6–84.0%)	
> 5	10/36	66.4% (44.6–81.2%)		6/18	57.0% (21.9–81.2%)		4/18	74.0% (44.6–89.4%)	
Clinical stage			0.256			0.391			0.389
II	12/53	80.3% (65.4–89.3%)		6/27	78.7% (55.9–90.6%)		6/26	82.1% (58.6–92.9%)	
III	23/82	73.3% (59.5–83.1%)		9/31	72.0% (41.1–88.5%)		14/51	73.5% (56.9–84.5%)	
cT			< 0.001*			0.748			< 0.001*
cT1/2/3	26/116	81.8% (72.1–88.3%)		13/54	78.5% (62.2–88.4%)		13/62	84.6% (71.4–92.0%)	
cT4	9/19	28.7% (5.0–59.4%)		2/4	50.0% (0.6–91.0%)		7/15	22.2% (1.4–58.8%)	
cN			0.758			0.086			0.199
cN0	8/36	79.3% (61.3–69.6%)		2/19	88.4% (60.8–97.0%)		6/17	70.1% (42.3–86.4%)	
cN+	27/99	74.5% (62.1–83.4%)		13/39	67.5% (43.2–83.2%)		14/60	78.4% (63.3–87.9%)	
Neoadjuvant treatment			0.414			0.410			–
CT	1/4	1.00		1/4	1.00		0/0	–	
CRT	36/140	77.1% (68.0–83.9%)		14/56	76.5% (60.1–86.8%)		22/84	77.6% (65.8–85.7%)	
Treatment complication			0.726			0.017*			0.308
Yes	5/21	69.2% (34.4%-88.1%)		4/9	0.00		1/12	88.9% (43.3–98.4%)	
No	32/123	78.6% (69.1–85.4%)		11/51	83.1% (67.3–91.7%)		21/72	75.8% (62.9–84.8%)	
Surgery			0.711			0.522			0.442
IL	20/84	78.1% (66.1–86.4%)		14/49	73.9% (56.4–85.3%)		6/35	84.0% (65.7–93.0%)	
McK	11/41	75.2% (55.9–87.0%)		0/4	1.00		11/37	73.5% (53.3–86.0%)	
TG	1/4	1.00		1/3	1.00		0/1	1.00	
Other	5/15	74.6% (39.8–91.1%)		0/4	1.00		5/11	68.6% (30.5–88.7%)	
Retrieved lymph node			0.924			0.464			0.566
< 15	6/30	77.5% (56.6–89.2%)		2/14	83.1% (47.2–95.5%)		4/16	73.3% (43.6–89.1%)	
≥ 15	30/111	76.5% (65.7–84.3%)		13/46	76.2% (57.2–87.6%)		17/65	76.9% (62.6–86.3%)	
Surgical complication			0.241			0.344			0.504
Yes	18/59	73.7% (57.9–84.3%)		6/24	80.1% (54.7–92.2%)		12/35	71.4% (50.5–84.7%)	
No	19/85	80.2% (68.6–87.9%)		9/36	77.4% (56.1–89.3%)		10/49	81.8% (66.7–90.5%)	
Major surgical complication			0.664			0.957			0.486
≥ 3°	12/37	68.9% (48.3–82.6%)		3/14	75.2% (40.7–91.4%)		9/23	67.0% (40.6–83.8%)	
< 3a	6/22	82.2% (52.5–94.2%)		3/10	90.0% (47.3–98.5%)		3/12	78.5% (36.1–94.5%)	

*AC* adenocarcinoma, *SCC* squamous cell carcinoma, *BMI* body mass index, *CCI* Charlson Comorbidity Index, *CRT* chemoradiotherapy, *CT* chemotherapy, *IL* Ivor Lewis, *McK* McKeown, *TG* total gastrectomy

Major surgical complications: ≥ IIIa according to Clavien-Dindo classification

*Statistically significant. The sum of patients for each column may not match the total due to some missing data

In multivariate analysis (Table 4), cT remained a significant predictor only in SCCs: the HR of cT4a/b cases with respect to cT1/cT2/cT3 cases was 8.13 (95% CI 2.04–32.37; *p* = 0.003). No significant predictive factors for recurrence were detected in ACs in the present series. Of note, sex and type of neoadjuvant treatment (chemoradiotherapy vs. chemotherapy) were excluded from multivariable analyses due to the low numbers of females and patients treated with neoadjuvant chemotherapy.

**Table 4:** Multivariate analysis for recurrence risk factors in pCR population *according by histotype.* Hazard ratios (HR) were derived by Cox regression model

Parameter	AC			SCC		
	HR	95% CI	*p* value	HR	95% CI	*p* value
Age (per 10 year increase)	1.08	0.48–2.43	0.875	0.74	0.28–1.92	0.541
BMI (overweight vs. normoweight)	4.28	0.53–34.43	0.171	2.19	0.46–10.28	0.318
cT (cT4 vs. cT1/cT2/cT3)	0.40	0.04–3.97	0.438	8.13	2.04–32.37	**0.003**
Treatment complication	3.39	0.70–16.25	0.127	0.64	0.07–5.37	0.683

*p* values were computed by the Wald test

*BMI* body mass index, *HR* hazard ratio, *CI* confidence interval

## Discussion

Pathological complete response after neoadjuvant chemoradiotherapy for EC represents the most desirable outcome—being associated with the best prognosis.^9^ However, evidence shows that also pCR patients relapse in 20–30% of cases.^6^,^10^ In our sample, recurrence occurred in 25.5% of pCR. In the present series, 5-year OS and CRS were 71.7% and 77.5%, respectively, and hence were slightly better than the values recorded in other recent reports.^3^,^5^,^6^,^15^,^16^

In the present study, we first examined the pattern of recurrence after pCR in terms of timing and site in both SCCs and ACs. Then, we analysed the predictive factors of recurrence that are the key elements to select postoperative tailored strategies and improve prognosis in such a selected cohort of patients as those with pCR after chemoradiotherapy.

With regard to the timing of recurrence, we found that ACs tend to relapse earlier compared to SCCs that have a slight tendency to recur after at least one year (Table 2b). Because neoadjuvant treatment is more effective in SCC cases, as observed in the CROSS trial,^1^ it could be hypothesized that neoadjuvant treatment delays the growth of SCC in some cases. The latest recurrence in our population was recorded 72 months after surgery, and it was a distant metastasis in SCC. Accordingly, Xi et al.^3^ and Steffen et al.^17^ found higher latency of systemic relapse in SCC than AC group, occurring even more than 4 years after surgery. For this reason, in agreement with some other authors,^18^ we highlight the need to extend surveillance to more than 5 years in SCCs even in case of pCRs. Another interesting finding of our series is that all the brain relapses occurred within 1 year from surgery, suggesting the need to include brain CT or MRI in preoperative imaging and early phase follow-up.

As for the site of relapse (Table 2a), all the recurrences in AC except one were systemic, whilst a third of relapses in SCC group occurred locally. The high rate of local relapses in SCCs after pCR raises the question of whether surgery for SCCs should include a routine extended mediastinal lymph node dissection in attempt to improve local control of disease as already hypothesized by some authors.^16^

Moreover, the high tendency of SCCs to recur locally despite pCR casts some doubts on the feasibility of watch and wait strategies that are currently widely considered for these tumors to avoid unnecessary esophagectomies.

However, all of the authors agree that the main challenge is represented by the systemic control of the disease even if a pCR is achieved. Due to a similar situation in rectal cancer, some evidence is recently emerging on the possible role of adjuvant chemotherapy on pCR patients.^19^ These results seem to encourage postoperative systemic therapy in selected categories of patients, with an advantage in terms of survival. In this scenario, there is the urgent need to identify a subpopulation of pCR who are at high risk of relapse and may therefore benefit from postoperative additional treatments.

In the present study, the analysis of predictive factors of recurrence after pCR showed that cT4a/b parameter at the initial staging is an independent risk factor for relapse in esophageal SCCs. Our findings are consistent with those reported by Chao et al.^15^ who studied a series of 70 SCCs achieving pCR after chemoradiotherapy and surgery and found that cT3-4 pretreatment stage increased five times recurrence risk compared with cT1-2. As mentioned, this finding is extremely important to identify pCR patients who may need additional postoperative chemotherapy.

In line with the most of previous reports, our study does not attribute any role to cN stage in predicting recurrence. This apparent paradox can be explained by the lower accuracy of the preoperative *N* staging compared to the T staging. Indeed, although the combination of EUS and CT allows to achieve great diagnostic accuracy in detecting nodal metastases (around 80%), the high percentage of false negatives (40–50%) limits the therapeutic usefulness of cN parameter.^20^,^21^

Some studies identified surgical complications as predictive factors for recurrence in pCR patients.^22^,^23^ These data were not confirmed by our analysis. Furthermore, complications of neoadjuvant treatment, tumor length, and number of lymph retrieved nodes were not independently associated with disease recurrence in our experience.

Interestingly, a new line of research identified the level of plasmatic circulating tumor DNA as a predictor of esophageal cancer development and recurrence after radical surgery.^24^ It could be interesting to analyse this association in SCCs, especially in pCR population. Indeed, this information may be added to the clinical risk factors for recurrences, such as cT stage, to better select patients who could benefit from adjuvant chemotherapy and stricter follow-up.

Unfortunately, no significant predictive factors for recurrence were detected in ACs in the present series. This may be related to the small sample size of ACs subgroup or may suggest that biomolecular factors, which were not considered in the present study, could play a role in predicting recurrences in ACs more than patients demographics (age, BMI) or stage-related tumor characteristics may have a role in predicting recurrences in ACs. However, even if no significant risk factors for recurrence was detected in ACs, we showed that nearly all the recurrences after pCR in ACs were systemic and occurred within 12 months, suggesting that early postoperative chemotherapy could play a central role in disease control of such patients.

The main limitation of the present study is the long-duration of the enrolment period. Indeed, during 18 years some variations in diagnostic accuracy, management of patients, and postoperative surveillance had occurred. However, the present is one of the largest available series in the west, and we do believe that the results of the present study may have a great clinical impact and represent an incentive for further research on this topic.

## Conclusions

Recurrence of EC after pCR is an unexpected event and represents a “hot topic” in the scientific community nowadays. Based on our experience, we can suggest that pCR patients with SCCs, who were cT4 at initial stage, should undergo adjuvant therapy and strict follow-up as a kind of precautionary principle. Our findings, together with other accumulating evidence, can contribute to develop appropriate tailored treatment, further improving prognosis in pCR patients.
